# Electroosmotic Mixing of Non-Newtonian Fluid in a Microchannel with Obstacles and Zeta Potential Heterogeneity

**DOI:** 10.3390/mi12040431

**Published:** 2021-04-14

**Authors:** Lanju Mei, Defu Cui, Jiayue Shen, Diganta Dutta, Willie Brown, Lei Zhang, Ibibia K. Dabipi

**Affiliations:** 1 Department of Engineering and Aviation Sciences, University of Maryland Eastern Shore, Princess Anne, MD 21853, USA; wlbrown@umes.edu (W.B.); lzhang@umes.edu (L.Z.); ikdabipi@umes.edu (I.K.D.); 2 Department of Computational Modeling and Simulation Engineering, Old Dominion University, Norfolk, VA 23529, USA; dcui001@odu.edu; 3 Department of Engineering Technology, SUNY Polytechnic Institute, Utica, NY 13502, USA; shenj@sunypoly.edu; 4 Department of Physics and Astronomy, University of Nebraska at Kearney, Kearney, NE 68849, USA; duttad2@unk.edu

**Keywords:** electroosmotic flow, micromixing performance, heterogeneous surface potential, wall obstacle, power-law fluid

## Abstract

This paper investigates the electroosmotic micromixing of non-Newtonian fluid in a microchannel with wall-mounted obstacles and surface potential heterogeneity on the obstacle surface. In the numerical simulation, the full model consisting of the Navier–Stokes equations and the Poisson–Nernst–Plank equations are solved for the electroosmotic fluid field, ion transport, and electric field, and the power law model is used to characterize the rheological behavior of the aqueous solution. The mixing performance is investigated under different parameters, such as electric double layer thickness, flow behavior index, obstacle surface zeta potential, obstacle dimension. Due to the zeta potential heterogeneity at the obstacle surface, vortical flow is formed near the obstacle surface, which can significantly improve the mixing efficiency. The results show that, the mixing efficiency can be improved by increasing the obstacle surface zeta potential, the flow behavior index, the obstacle height, the EDL thickness.

## 1. Introduction

In recent decades, microfluidics has attracted significant attention with its increasing applications in chemical synthesis, biomedical analysis, drug delivery [1,2,3]. Mixing of species in a microfluidics plays an important role in many of these applications. However, the small scale of microfluidic system leads to a low Reynolds number and laminar flow behavior. The mixing under this situation becomes difficult and, thus, efficient mixing mechanism is a great demand in microfluidic devices [4,5,6,7].

Micromixers can be categorized into passive and active micromixers, depending on the actuation mechanism. Active micromixers require use of external energy source, such as pressure [8], acoustics [9], electric field [10], and magnetic field [11]. Passive micromixers, on the other hand, utilize surface structure modification, obstacles or grooves to enhance the mixing of the fluids. Compared to active micromixers, passive micromixers do not need active moving parts and are easier on fabrication and operation [4,12]. Among various passive mixing strategies, electroosmotic flow (EOF), with its flexibility of adjusting the flow patterns by manipulation of surface properties and geometry, is widely used to enhance the mixing performance in microfluidics [6,13,14]. A number of theoretical and experimental studies have been done to improve the mixing efficiency in microchannels by proper design of surface zeta potential [15], surface topology [16], and geometrical configuration [17], etc. Basati et al. [18] investigated the effect of zeta potential distribution and geometrical specifications on the mixing performance of EOF in converging-diverging microchannels. Bhattacharyya et al. [19] studied the vortex formation of combined pressure-driven EOF in a microchannel with a rectangular obstacle on the wall. Wang et al. [20] numerically investigated the vortex formation near a two-part cylinder under an external DC electric field. Chen et al. [21] presented a novel electroosmotic micromixer that consists of arrays of asymmetric electrodes and lateral which can enhance mixing efficiency with applied potential. Seo et al. [22] studied the mixing characteristics in straight microchannel with various obstacle configuration and concluded that the rectangular obstacle shows the most effective mixing enhancement. Many of these theoretical studies on the electrokinetic micromixing in the literature assume Newtonian fluid behavior. However, the biomedical and chemical applications often involve the use of complex solutions (e.g., polymer solution, blood) which exhibit non-Newtonian characteristics. Understanding the mixing performance of the EOF for non-Newtonian fluids is important for the experimental design of efficient micromixers. Various non-Newtonian models have been used to characterize the rheological behavior of the electrokinetically driven complex solution, such as the Carreau–Yasuda model [23], power-law model [24], Oldroyd-B model [25], and generalized Maxwell model [26].

In recent past, several numerical and analytical studies have been performed to investigate the electroosmotic mixing of non-Newtonian fluid in rectangular, cylindrical, and wavy microchannels. To describe the electric potential within the electric double layer (EDL) near the charged surface, the Boltzmann distribution [25,27,28] or the Smoluchowski slip velocity boundary condition [29,30] is commonly used in these studies. Compared to the general Nernst–Planck model, the use of Boltzmann distribution or Smoluchowski slip velocity boundary can reduce the computational effort, but has some limitations [31,32,33]. On the frame of Nernst–Planck theory, Banerjee et al. numerically investigated the electrokinetic micromixing of power-law fluid both in cylindrical microchannels with surface contraction/expansion [34] and in a wavy patterned microchannel with sinusoidal zeta potential distribution [35]. Mei et al. [36] investigated the EOF of Linear Phan–Thien–Tanner (LPTT) fluid in a nanoslit. To the best knowledge of the authors, on the electroosmotic mixing of a power-law fluid in straight microchannels with rectangular obstacle and surface potential heterogeneity, no study has been done using the Nernst–Planck theory. The mixing in rectangular microchannels is of importance as it can provide very useful information on the design of efficient T/Y-micromixers.

In this study, the full model consisting of Navier–Stokes and Poisson–Nernst–Plank equations is considered to analyze the mixing performance in the microchannel with rectangular obstacle and surface potential heterogeneity. The power-law model is used for non-Newtonian fluid due to its simplicity and the ability to characterize the rheological behavior of non-Newtonian fluids [37]. The paper is organized as follows. In Section 2, the mathematical model describing the electroosmotic mixing of power-law fluid in the microchannel is presented. Section 3 presents the numerical calculation details and validation of our numerical results. In Section 4, effects of the heterogeneous zeta potential, the flow behavior index, the obstacle dimension, and the EDL thickness on the mixing performance of the microchannel are examined in detail. Section 5 concludes the paper.

## 2. Mathematical Model

**Figure 1:**
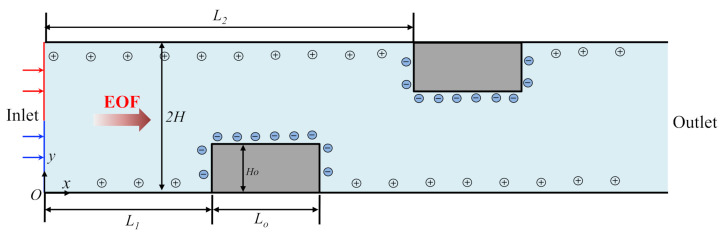
Schematic diagram of the EOF in the microchannel with wall-mounted rectangular obstacles and heterogeneous zeta potential. The EOF is induced by an external potential bias *V*_0_ acting across the channel.

Figure 1 illustrates the schematic diagram of the 2D microchannel filled with incompressible KCl electrolyte solution that is driven by an external potential bias *V*_0_ acting along the streamwise direction across the channel. The channel is of height 2H and length L, with two obstacles of height $H_{o}$ and length $L_{o}$ mounted on the lower and upper wall of the channel. The obstacles are located at a distance of $L_{1}$ and $L_{2}$ from the inlet, respectively. The channel wall is assumed to be distributed with constant negative zeta potential $\zeta_{c}$, except on the obstacle surface, where oppositive zeta potential $\zeta_{w}$ is distributed to create surface potential heterogeneity. Two fluid streams containing uncharged sample species of different concentration are injected at the inlet of channel, represented by red and blue arrow/line, respectively. As the fluid flows downstream, the uncharged sample species within these two fluid streams are gradually mixed. Cartesian coordinate system O-*xy* is adopted with *x*-axis in the length direction, *y*-axis in the height direction, and the origin fixed on the bottom corner at the channel inlet.

### 2.1. Governing Equations

The steady-state transport of the non-Newtonian electrolyte solution induced by the external electric field is governed by the mass and momentum conservation equation as:

(1) ∇·u=0,

(2) $$\rho u\cdot \nabla u=-\nabla p+\nabla \cdot \left(2\mu \Gamma \right)-\rho_{e}\nabla \Phi .$$

where u is the velocity field; *p* denotes the pressure; Φ is the electric potential, and *ρ_e_* is the volume charge density within the electrolyte solution; *ρ* represents the fluid density; $\Gamma =\left[\nabla u+{\left(\nabla u\right)}^{T}\right]/2$ is the strain rate tensor. The viscosity of the fluid is given by $\mu =m{\left(\Gamma \right)}^{n-1}$ for a power-law fluid, where m is the flow consistency index, n is the flow behavior index, and $\Gamma =\sqrt{\Gamma :\Gamma }$ is the magnitude of the shear rate tensor. It is noted that the shear thinning fluid, Newtonian fluid, and shear thickening fluid correspond to n<1, n=1, and n>1, respectively.

The charged channel surface in contact with the electrolyte solution will develop an electric double layer (EDL) enriched with counterions in the vicinity of the charged surface. The electric potential distribution is determined by the superposition of the external electric potential ψ and induced electric potential ϕ (due to EDL). The electric potential and ion transport within the electrolyte solution are governed by the Laplace equation, Poisson equation, and the Nernst–Planck equation as:

(3) $$-\varepsilon_{f}\nabla^{2}\psi =0$$

(4) $$-\varepsilon_{f}\nabla^{2}\emptyset ＝F\left(c_{1}-c_{2}\right)$$

(5) $$\nabla \cdot \left(uc_{i}-D_{i}\nabla c_{i}-z_{i}\frac{D_{i}}{RT}Fc_{i}\nabla \emptyset \right)=0,\;i=1,\;2,$$

In the above, $\varepsilon_{f}$ is the permittivity of the electrolyte solution; $z_{i}$, $D_{i}$, and $c_{i}$ are the valence, diffusivity, and ionic concentration of ionic species K^+^ (*i* = 1) and Cl^−^ (*i* = 2), respectively; F, *R*, and *T* are the Faraday constant, gas constant, and absolute temperature, respectively.

The governing equation for the concentration of the uncharged sample species can be obtained from Equation (5), with the corresponding valance set to 0, which results in the convection-diffusion equation as:

(6) $$\left(u\cdot \nabla \right)C-D\nabla^{2}C=0$$

where C represents the concentration of the uncharged species, and *D* denotes its diffusivity.

### 2.2. Dimensionless Equations

The dimensionless form of the governing equations is derived in the following. Select the half of the channel height *H* as length scale, the EOF velocity under constant viscosity $u_{0}=\varepsilon_{f}R^{2}T^{2}/\left(\mu_{0}HF^{2}\right)$ as velocity scale, the constant viscosity $\mu_{0}$ is the viscosity at $\Gamma =1\;s^{-1}$ and has the same magnitude as m, $\rho u_{0}{}^{2}$ as the pressure scale, the thermal potential *RT*/*F* as electric potential scale, the bulk concentration of the KCl electrolyte *C*_0_ as the ionic concentration scale, the set of governing Equations (1)–(6) can be normalized as:

(7) $$\nabla^{\prime }\cdot u^{\prime }=0,$$

(8) $$u^{\prime }\cdot \nabla^{\prime }u^{\prime }=-\nabla^{\prime }p^{\prime }+\frac{1}{Re}\nabla^{\prime }\cdot \left(2\mu^{\prime }\Gamma^{\prime }\right)-\frac{\mu_{0}^{\prime }{\left(kH\right)}^{2}}{2Re}\left(c_{1}^{\prime }-c_{2}^{\prime }\right)\nabla^{\prime }\left(\emptyset^{\prime }+\psi^{\prime }\right),$$

(9) $$\nabla^{2}\psi^{\prime }=0,$$

(10) $${\nabla^{\prime }}^{2}\emptyset^{\prime }=\frac{1}{2}{\left(kH\right)}^{2}\left(c_{1}^{\prime }-c_{2}^{\prime }\right)$$

(11) $$\nabla^{\prime }\cdot \left(u^{\prime }c_{i}^{\prime }-\frac{D_{i}}{Hu_{0}}\nabla^{\prime }c_{i}^{\prime }-\frac{z_{i}D_{i}}{Hu_{0}}c_{i}^{\prime }\nabla^{\prime }\emptyset^{\prime }\right)=0,\;i=1,2.$$

(12) $$\left(u^{\prime }\cdot \nabla^{\prime }\right)C^{\prime }-\frac{D}{Hu_{0}}{\nabla^{\prime }}^{2}C^{\prime }=0$$

In the above, all variables with prime indicate their dimensionless form; the Reynolds number is $=\rho u_{0}^{2-n}H^{n}/m$; the dimensionless viscosity constant $\mu_{0}^{\prime }=\frac{\mu_{0}}{mu_{0}^{n-1}H^{1-n}}$; the Debye length is $\lambda_{D}=\frac{1}{k}=\sqrt{\varepsilon_{f}RT/\overset{2}{\underset{i=1}{\sum }}F^{2}z_{i}^{2}C_{0}}$; the dimensionless viscosity is $\mu^{\prime }={\left(\Gamma^{\prime }\right)}^{n-1}$.

### 2.3. Boundary Conditions

To solve for the coupled differential Equations (7)–(12), the boundary conditions are set as following.

On the channel wall, non-slip and no-ion penetration boundary condition is applied as:

(13) $$u^{\prime }=0,\;n\cdot \nabla^{\prime }\psi^{\prime }=0,\;\emptyset^{\prime }=\zeta ,\;n\cdot \left(-\nabla^{\prime }c_{i}^{\prime }-z_{i}c_{i}^{\prime }\nabla^{\prime }\emptyset^{\prime }\right)=0,\;n\cdot \nabla^{\prime }C^{\prime }=0$$

where $\zeta =\zeta_{c}$ on the channel wall, and $\zeta =\zeta_{w}$ on the obstacle surface, n represents the normal unit vector on the surface.

At the inlet, stress free boundary condition is applied, concentration of the KCl is set as the bulk concentration, and the concentration of the uncharged species follows a step-like concentration distribution, as:

(14) $$n\cdot \nabla^{\prime }u^{\prime }=0,\;\;p^{\prime }=0,\;\psi^{\prime }=V_{0}\cdot \frac{F}{RT},\;\;\emptyset^{\prime }=0,\;c_{1}^{\prime }=c_{2}^{\prime }=1,\;C^{\prime }=\left\{\begin{array}{c} 1,\;y^{\prime }>1\\ 0,\;y^{\prime }\leq 1\; \end{array}\right.$$

At the outlet, stress free boundary condition is applied, and the electric potential is set as 0:

(15) $$n\cdot \nabla^{\prime }u^{\prime }=0,\;\;p^{\prime }=0,\;\;\psi^{\prime }=0,\;\emptyset^{\prime }=0,\;c_{1}^{\prime }=c_{2}^{\prime }=1,\;\frac{\partial C^{\prime }}{\partial x\prime }=0$$

## 3. Numerical Method and Validation

**Figure 2:**
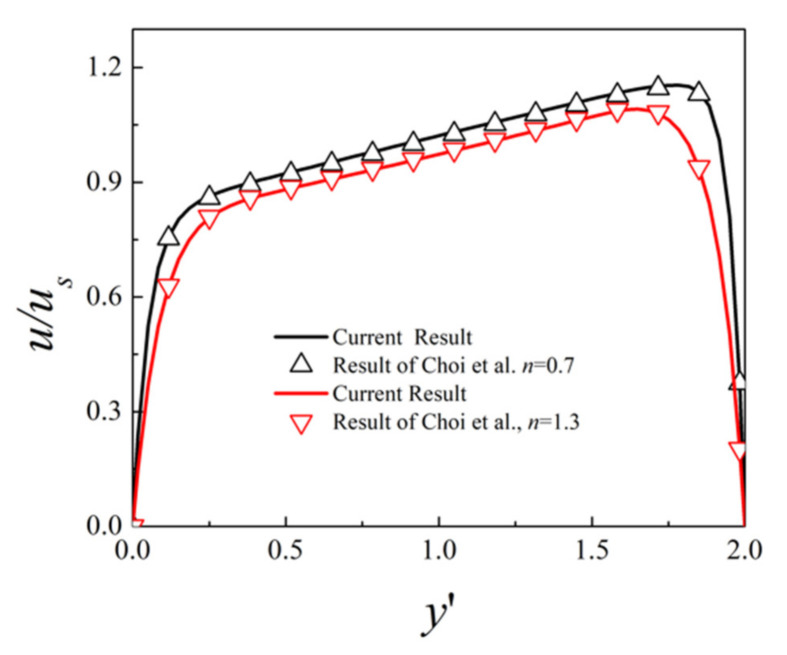
Dimensionless velocity distributions $u/u_{s}$ within the microchannel of asymmetric zeta potentials on the walls for fluid behavior index *n* = 0.7 and *n* = 1.3: lines (current simulation result) and symbols (analytical result of Choi et al. [38].

The coupled Equations (7)–(12) along with the boundary conditions (13)–(15) are numerical solved using the commercial finite element software COMSOL Multiphysics (version 5.1) with its AC/DC module, CFD module, chemical reaction engineering module, and MUMPS solver. As the flow field and electric field have larger variation within the EDL, finer mesh is distributed near the channel surface and the obstacle surface, and mesh independence study is carried out to ensure the accuracy of the simulation. To further validate the accuracy of the current simulation, we compare our simulation result with Choi et al. [38] who derived the analytical solution of EOF velocity of power-law fluids in a slit microchannel with asymmetric zeta potentials at top and bottom walls. The parameters are set as *V*_0_ = 1.5 V, kH=15 $D_{1}\left(D_{2}\right)=1.96\;\left(2.03\right)\times 10^{-9}m^{2}s^{-1}$, $\varepsilon_{f}=7.08\times 10^{-10}{\mathrm{CV}}^{-1}m^{-1}$, 2H=10 μm, L=30H, $m=10^{-3}\mathrm{Pa}\cdot s^{n}$, $F=96,485\;C\cdot {\mathrm{mol}}^{-1}$, $R=8.314\;J\cdot {\mathrm{mol}}^{-1}K^{-1}$, T=298 K, zeta potential ζ=−10 mV at the bottom surface and ζ=−15 mV at the top surface. The profile of the dimensionless mainstream velocity component along the middle line $x^{\prime }=15$ for different fluid behavior index *n* is plotted in Figure 2. Here the velocity is scaled by $u_{s}=nk^{\frac{1}{n}-1}{\left(\frac{\;\varepsilon_{f}V_{0}\zeta_{m}}{mL}\right)}^{\frac{1}{n}}$ with $\zeta_{m}$ being the average zeta potential of the top and bottom surface zeta potential. The results show that the velocity increases rapidly near the wall within the EDL, and the gradient of velocity is larger near the top surface due to the larger zeta potential. The dimensionless velocity decreases with increasing fluid behavior index *n*, due to the overall increased viscosity. It can be seen that the EOF velocity for power-law fluid under asymmetric zeta potential from the current simulation matches well with the analytical solution of Choi et al. [38]. In the following simulations, the parameters are set as $V_{0}=1\;V$, $kH=10,\;L=20H,\;L_{1}=6H,\;L_{2}=10H,\;L_{o}=2H,\;H_{o}=0.2H,\;\zeta_{c}=-20\;\mathrm{mV}$, $\zeta_{w}=20\;\mathrm{mV}$, other parameters are set as mentioned above unless otherwise specified.

To characterize the mixing performance within the microchannel, the mixing efficiency of the uncharged species is defined as:

(16) $$\eta \left(x^{\prime }\right)=\left[1-\frac{\int_{y^{\prime }\_bottom}^{y^{\prime }\_top}\left|C^{\prime }-C_{\infty }\right|dy^{\prime }}{\int_{y^{\prime }\_bottom}^{y^{\prime }\_top}\left|C_{0}-C_{\infty }\right|dy^{\prime }}\right]\times 100\%,$$

where $C_{\infty }=0.5$ and $C_{0}=0\;\left(or\;1\right)$ are the fully mixed concentration and totally unmixed concentration, respectively.

## 4. Results and Discussion

### 4.1. Effect of Obstacle Surface Zeta Potential

**Figure 3:**
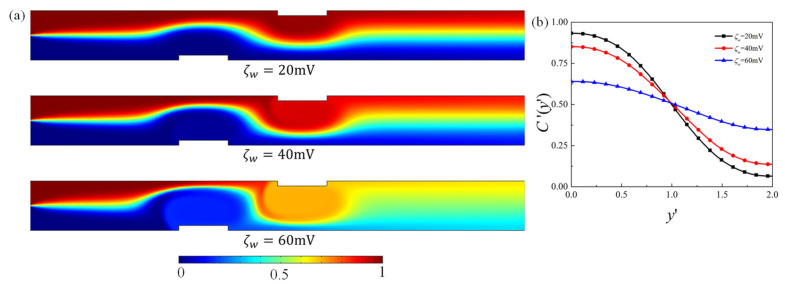
(**a**) Contour plot of elute species concentration $C^{\prime }$ in the microchannel, (**b**) distribution of species concentration at the outlet of the microchannel, for different obstacle surface zeta potential $\zeta_{w}=20\;\mathrm{mV}$, 40 mV, and 60 mV.

**Figure 4:**
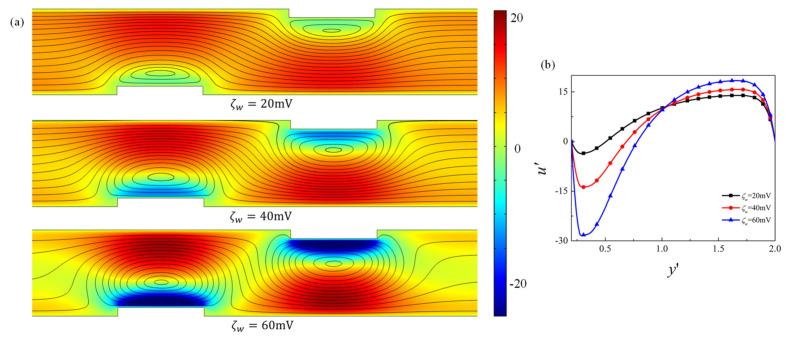
(**a**) Velocity contour and streamlines for different obstacle surface zeta potential, (**b**) mainstream velocity component $u^{\prime }$ along the cross section located at the center of the first obstacle $x^{\prime }=7$, for $\zeta_{w}=20\;\mathrm{mV}$, 40 mV, and 60 mV.

**Figure 5:**
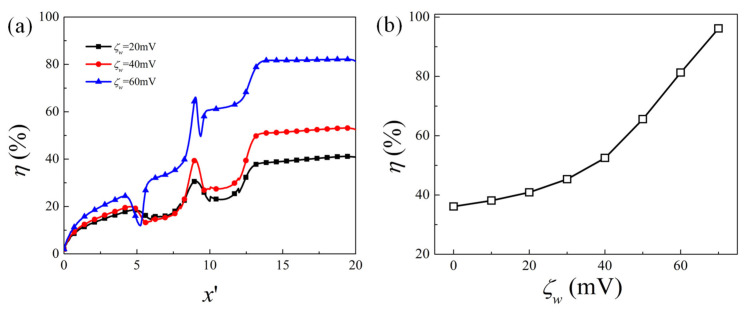
(**a**) Evolution of the mixing efficiency along the channel length direction for different obstacle surface zeta potential; (**b**) the variation of mixing efficiency at the outlet with obstacle surface zeta potential $\;\zeta_{w}$.

First of all, the effect of the obstacle surface zeta potential on the mixing performance for the fixed geometry is examined. Figure 3 presents the contour plot of the elute species concentration C′ in the microchannel and the concentration profile at the channel outlet for obstacle surface zeta potential $\zeta_{w}=20\;\mathrm{mV}$, 40 mV, and 60 mV, respectively. For higher heterogeneous zeta potential $\zeta_{w}$, significant improvement of mixing is achieved after the fluid flows past the obstacle. The distribution of concentration C′ at the outlet shows that the species approaches uniform distribution as $\zeta_{w}$ increases, revealing better mixing performance. The corresponding velocity contour and flow streamlines are plotted in Figure 4 to analyze the effect of the obstacle and zeta potential on the flow field. It can be observed that in the straight part away from the obstacles, streamlines are parallel to the channel surface. In the region where the obstacle is present, the streamlines are distorted and vortex is formed in the vicinity of the obstacle surface. The positive zeta potential at the obstacle surface induces the negative mainstream velocity near the surface, which in turn results in the vortex formation. The velocity profile along the cross-sectional line located at the center of the first obstacle (i.e., $x^{\prime }=7$) is shown in Figure 4b. It shows that as the magnitude of the zeta potential at the obstacle surface increases, the backward velocity near the surface becomes much larger, the vortex becomes stronger and the vortex center moves towards the centerline of the microchannel, which contributes to the better mixing performance of the elute species. Figure 5 further plots the variation of the mixing efficiency along the channel length direction and the dependency of the mixing efficiency at the outlet on the obstacle surface zeta potential $\zeta_{w}$. It clearly shows that at the fixed zeta potential $\zeta_{w}$, significant improvement of the mixing efficiency occurs right after the fluid flows past each obstacle. The mixing efficiency at the channel outlet monotonously increases with the heterogeneous zeta potential. The mixing efficiency at the outlet for $\zeta_{w}=70\;\mathrm{mV}$ is 2.7 times that of $\zeta_{w}=0\mathrm{mV}$.

### 4.2. Effect of Flow Behavior Index

**Figure 6:**
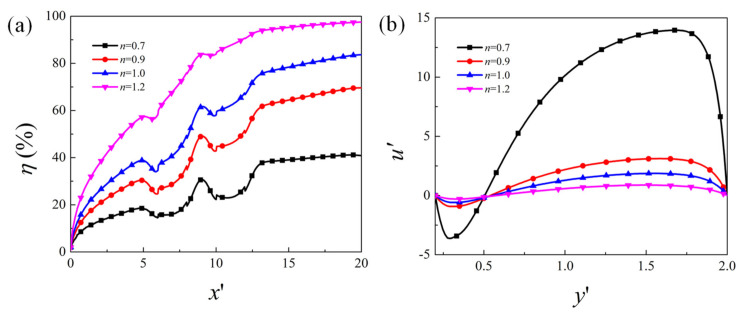
(**a**) Evolution of the mixing efficiency along the channel length direction for different flow behavior index *n*; (**b**) mainstream velocity component $u^{\prime }$ along the cross section located at the center of the first obstacle $x^{\prime }=7$.

Figure 6 presents the mixing efficiency along the channel length direction and mainstream velocity component $u^{\prime }$ along the cross-section located at the center of the first obstacle ($x^{\prime }=7$) for various flow behavior index *n*. It is obvious that the mixing efficiency becomes much higher for larger value of *n*. Under the same condition, the shear thickening fluid has better mixing performance than the Newtonian fluid (*n* = 1), and the shear thinning fluid has lower mixing efficiency. The mainstream velocity component is negative near the surface of the obstacle due to the positive zeta potential, and is positive within a large portion of the channel. The velocity varies more steeply near the wall and the overall velocity magnitude is much larger for a smaller value of *n*. The dimensionless flow rate $Q^{\prime }=\int_{0.2}^{2}u^{\prime }dy^{\prime }$ is 14.4, 3.0, 1.8, and 0.8 for *n* = 0.7, 0.9, 1.0, and 1.2, respectively. The result is consistent with that from Banerjee et al. [34]. This is because, under the fixed shear rate, the viscosity for the power-law fluid is larger for higher *n*, which results in the lower velocity under the same electric condition. When the velocity is smaller, the solute species can get more diffusion flux to improve downstream mixing and, thus, better mixing performance is achieved for higher *n*.

### 4.3. Effect of Obstacle Height

**Figure 7:**
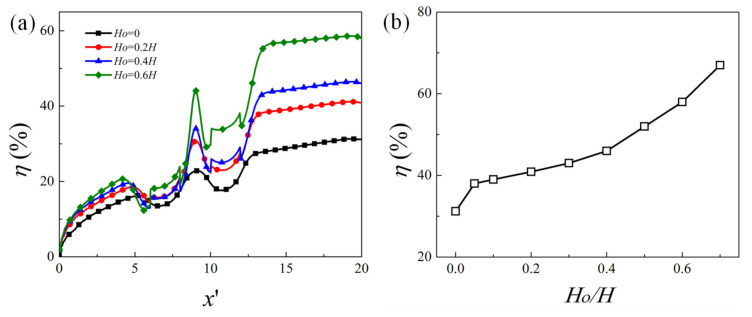
(**a**) Evolution of the mixing efficiency along the channel length direction for obstacle height $H_{o}=0,\;0.2H,\;0.4H,\;\mathrm{and}\;0.6H$, (**b**) the dependence of the mixing efficiency at the outlet as a function of ratio $H_{o}/H$.

The effect of the obstacle height on the mixing efficiency is presented in Figure 7, where the evolution of the mixing efficiency along the channel length direction and the variation of mixing efficiency at the outlet as a function of the obstacle height $H_{o}$ are plotted. It can be seen that as the obstacle height becomes larger, the mixing performance is better. Compared to the microchannel without obstacle, the presence of the obstacle can improve the mixing performance very effectively. The mixing efficiency at the outlet for $H_{o}=0.7H$ is 2.2 times of that without obstacle (i.e., $H_{o}=0$).

### 4.4. Effect of EDL Thickness

**Figure 8:**
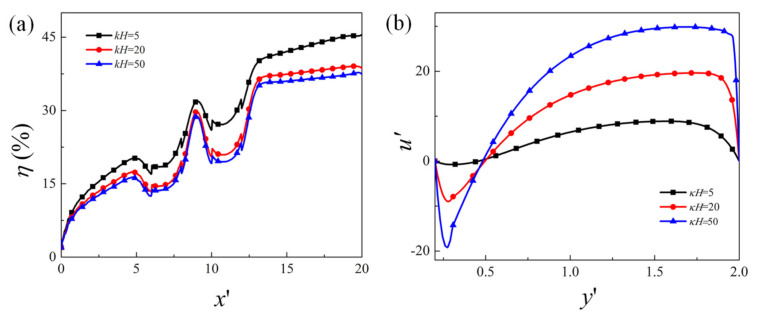
(**a**) Evolution of the mixing efficiency along the channel length direction, (**b**) mainstream velocity component $u^{\prime }$ along the cross-section located at the center of the first obstacle $x^{\prime }=7$ for EDL thickness: kH=5, 20, and 50.

Finally, the effect of EDL thickness on the mixing performance is presented in Figure 8, where the variation of mixing efficiency along the channel length direction for EDL thickness kH=5, 20, and 50 is plotted. It can be seen that the mixing efficiency is slightly higher for larger EDL thickness (i.e., kH=5) than that of thin EDL thickness (i.e., kH=20 and 50). As shown in the cross-sectional mainstream velocity profile in Figure 8b, when the EDL thickness is comparable to the channel height, which is true when the electrolyte concentration is low, the change of the velocity near the wall is small, and the overall velocity is relatively small. This means that the vortex near the obstacle surface is weaker. When the EDL is very thin (e.g., kH=50), the gradient of the velocity is very large in the vicinity of the wall, and the velocity is much larger than that of the large EDL thickness. The effect of EDL thickness on the EOF velocity is consistent with that in the literature, and has been well explained [36,39]. When the velocity is smaller, the diffusion effect becomes stronger, which results in a slight increase in the mixing efficiency.

## 5. Conclusions

In this study, the steady-state mixing performance of electroosmotic flow of the power-law fluid is numerically investigated in a 2D microchannel with wall mounted obstacles and heterogeneous zeta potential. The numerical simulation is based on the full model consisting of the Poisson–Nernst–Planck and Navier–Stokes equations. Compared to the channel without obstacle, the presence of the obstacle can significantly increase the mixing efficiency. By increasing the obstacle height, the mixing efficiency can be further improved. The heterogeneous zeta potential on the obstacle surface induces vortical flow in the vicinity of the obstacle surface, and the vortex strength becomes stronger as the zeta potential increases, which results in the improvement of the mixing performance. Additionally, for larger behavior index of the power-law fluid, velocity becomes smaller, the transport of the uncharged species becomes diffusion dominant, resulting in better mixing performance. For relatively large EDL thickness, the variation of velocity near the surface is smaller, and the mixing efficiency is slightly higher than that of thin EDL thickness due to the overall lower velocity.
